# Medical artificial intelligence and the right to health

**DOI:** 10.1186/s12910-026-01536-x

**Published:** 2026-06-29

**Authors:** Sinead Prince, Serene Ong, Kathryn Lynn Muyskens, Julian Savulescu, Sebastian Porsdam Mann

**Affiliations:** 1 Centre for Biomedical Ethics, Yong Loo Lin School of Medicine, National University of Singapore, Singapore, Singapore; 2 Uehiro Oxford Institute, University of Oxford, Oxford, UK; 3 Faculty of Law, Center for Advanced Studies in Bioscience Innovation Law, University of Copenhagen, Copenhagen, Denmark

**Keywords:** Human rights, Health, Artificial intelligence, Healthcare

## Abstract

The international human right to health states that people have claims to access goods, services, and infrastructure that protects an adequate standard of health. The current and forthcoming advances in artificial intelligence (AI), which significantly improve the quality and efficacy of medical care, could raise the current standard of healthcare owed under the right to health. In this paper, we identify three duties owed under the right to health and describe ways in which AI can uniquely or more efficiently fulfill these duties, namely the duties to provide: access to good quality care that effectively improves patient health; equitable access to healthcare for all persons; and novel healthcare services that can only be achievable by medical AI. This paper will also consider some potential objections to our claim such as whether medical AI may undermine, rather than fulfil, the social determinants of health. We conclude that if medical AI can partially fulfil the right to health through increasing access and quality of healthcare, and if providing medical AI can be balanced against other obligations and rights, states will have corresponding duties to not unnecessarily restrict AI innovation through regulation and may even be required to invest in medical AI infrastructure to meet the right to health of their populations.

## Introduction

The invention of artificial intelligence (AI) and its significance in healthcare raises questions about the relationship between AI and human rights, as reflected in international human rights law. AI is generally defined as technology, such as computer systems, that can simulate human intelligence, namely by processing data and information from various sources [1]. The various kinds of current technologies under the AI umbrella vary from limited and narrow algorithms with simple decision-trees, such as facial detection and recognition, to generative AI, such as Large Language Models (LLMs; e.g., ChatGPT). Within healthcare, AI can perform some tasks currently undertaken by doctors, such as performing diagnostics, prognostics, and providing access to health information [2, 3]. AI can also help reduce healthcare providers’ burden in administrative tasks [4]. Generative AI models, in particular, show promise in a wide variety of associated tasks, such as augmenting consent processes [5] and improving the capture of patients’ qualitative experience [6]. AI can help facilitate health research by improving the design of clinical trials [7], facilitating ethics review [8, 9], and summarizing, editing, and generating text for use in medical publications [10].

Discussions about AI and human rights generally revolve around how human rights frameworks can best *regulate* AI [11–15]. For example, international human rights treaties recognize the human right to privacy, and therefore States parties to these treaties are subject to legal obligations to regulate AI in ways that protect patient privacy [11]. While AI is often seen as a potential transgressive force in relation to human rights, the use of AI may also positively affect the fulfilment of existing rights.^1^ The invention of new technologies and tools can create the need to both revise existing rights and introduce new rights. It can also raise the standards for addressing these rights. By analogy, the invention of vaccines renders their use a standard tool in fulfilling a person’s right to health. For this reason, Ho [19] has claimed that alongside the right to science, there is a duty of states to make available and accessible AI that maximizes patient health. Echeverría [20] has also surveyed the variety of medical AI tools that can improve human health, and has identified the ways in which AI could undermine the social dimension of the human right to health, particularly through inaccuracy, inexplicability, and threats to privacy. Following this burgeoning research, we further these claims and argue whether access to medical AI may be *necessary* to partially fulfil the human right to health.

This paper argues that governments have corresponding duties not to unnecessarily restrict AI innovation through regulation and may even be required to invest in medical AI infrastructure to meet the right to health of their populations. We will argue that such duties arise when medical AI significantly impacts healthcare in either of two main ways. First, when medical AI significantly increases the efficiency of healthcare services (‘efficient AI’), such as AI that streamlines administrative processes [4], automates medical imaging diagnoses [21], and triages patients in emergency wards [22]. Second, when medical AI creates novel solutions to healthcare problems that cannot be resolved through other means (‘novel AI’), such as precision medicine [23] or predicting adverse physical and mental health outcomes, for example via facial recognition technologies [24]. We limit this claim according to the requirement of States to progressively realize the right to health, and acknowledge that insofar as the benefits of efficient AI can be equally achieved by other means (such as by funding priorities), many States will be justified in prioritizing other rights, and other means of fulfilling the right to health. Furthermore, our arguments are not meant to imply that provision of AI should be prioritized over basic requirements, such as those of safe food and water. Consequently, much of our analysis will apply to high-income countries. However, the fact that some people’s rights to other goods in low and middle income countries are not met ought not prevent us from discussing the obligations flowing from the right to health in light of the evolving technologies. More importantly, our arguments apply relative to the resources available in those countries.^2^ It is often both possible and necessary to address multiple problems at once. Nevertheless, to the extent that investing in such AI will cost-effectively, efficiently, and beneficially improve healthcare access and outcomes, our thesis means that many States that have capacity to invest in efficient and novel medical AI will have derivate duties under the right to health to do so.

To make these arguments, we will first briefly define the right to health and its normative environment in international human rights law (Section II). We next present an analysis of the ways in which efficient AI and novel AI can significantly improve access to healthcare and healthcare outcomes and argue that such transformative technologies are required to partially fulfill the human right to health (Section III). We then consider potential objections to this claim (Section IV), and provide some comments about how to proportionally balance claims about medical AI with other human rights (Section V). We conclude with analysis of the implications of these arguments for the obligations of States parties under treaties recognizing the right to health in both providing and regulating medical AI (Section V).

## The right to health

To properly frame the arguments that follow, it is necessary first to situate this paper in the literature surrounding the human right to health more generally. Human rights have both moral and legal dimensions, and the philosophical literature continues to debate what makes for the best theoretical grounding.^3^ For the arguments that follow, we start from the understanding that human rights are legal and political instruments with two primary functions, first to express the minimum moral obligations owed to each human being, and second, as mechanisms of generating accountability beyond the nation state [33, 34]. In line with this, we take international documents like Article 25.1 of the *Universal Declaration of Human Rights* (UDHR), Article 12 of the *International Covenant on Economic, Social and Cultural Rights* (ICESCR) [35], as well as expert treaty body interpretations of the right as expressed in the UN Committee on Economic, Social and Cultural Rights’ (CESCR’s) General Comment 14 as our starting point [36]. These expert treaty body pronouncements are not legally binding but are widely considered to carry considerable weight [37–39].

Generally, debate over the right to health has focused on questions about its scope and limits.^4^ If we recognize the universal value of a particular claim such as the right to health, what is the exact nature of the duty owed? The foundational, although not legally binding, UDHR [43] Article 25 states that “everyone has the right to a standard of living adequate for the health and well-being” for themselves [44, 45]. Article 25 recognizes the determinants for achieving this standard of living: adequate “food, clothing, housing and medical care and necessary social services, and the right to security in the event of unemployment, sickness, disability, widowhood, old age or other lack of livelihood in circumstances beyond his control.” The legally binding treaty based on the UDHR is the ICESCR [35], which is the primary source of State obligations in relation to health under international humans rights law. Article 12 of the ICESCR recognizes “the right of everyone to the enjoyment of the highest attainable standard of physical and mental health.” The ICESCR also recognizes some necessary measures of achieving this right; of note is the “prevention, treatment and control of epidemic, endemic, occupational and other diseases” and “[t]he creation of conditions which would assure to all medical service and medical attention in the event of sickness.” In General Comment 14 [36], the UN CESCR explored and defined the purpose and scope of the right to health. General Comment 14 focused heavily on the determinants of health: including the individual’s biological and socio-economic preconditions and the State’s resources. Thus, “[c]onsequently, the right to health must be understood as a right to the enjoyment of a variety of facilities, goods, services and conditions necessary for the realization of the highest attainable standard of health.” The right to health is therefore generally understood in international law as the right to have one’s State invest in infrastructure, personnel, services, and goods that directly protect the determinants of human health and is thus broader than the right of access to suitable healthcare.

There is nevertheless tension between the idealistic language of “highest attainable standards” and the obvious constraints imposed by limited resources and other practical realities. In recognition of this, General Comment 14, as well as the general implementation clause in Article 2 [1] ICESCR, employ language that emphasizes the need for ‘progressive realization’. Specifically, General Comment 14 describes progressive realization as States parties having the “specific and continuing obligation to move as expeditiously and effectively as possible towards the full realization of article 12.” The international community recognizes that not all states are able to synchronously address the right to health. The infrastructure and systems to protect public health (ie., such as safe water, food security, trained personnel, and efficient healthcare systems) take years to complete [46]. Therefore, the right to health is *not* the right to be healthy, but the right to have one’s State “move as expeditiously and effectively as possible towards the full realization’ of the right to health” [36]. For example, States cannot promise perfect and affordable healthcare for all conditions and illnesses, but they can offer good quality resources across the most common illnesses and conditions as and when they can afford them. Indeed, some treatments might be so expensive and useful for such a small minority of the population, or treat not sufficiently serious conditions, that providing such treatments would fail to be cost-effective and be unfair to expect as per the right to health.

In this context, two claims are relevant to medical AI. First, recent advances suggest that AI could enable entirely novel forms of treatment or prevention, as seen in AI-enabled research breakthroughs such as AlphaFold’s protein structure prediction for drug design [47], deep learning–driven antibiotic discovery [48], and generative AI for drug design and optimization [49]. While these breakthroughs are early-stage, they illustrate the trajectory of AI research and its potential to yield treatments and preventive measures that were previously out of reach. Second, empirical evidence demonstrates that certain medical AI applications already yield substantial time or cost savings—even if a comprehensive cost-effectiveness analysis lies beyond our current aims. For instance, AI-assisted diagnostics and treatment workflows have the potential to produce significant economic benefits [50]. At scale, AI-enabled hospital operations could save between 5 and 10% of total costs—translating to an estimated US$60 billion to US$120 billion in annual net savings within five years [51], and predictive AI tools of patient discharge and intensive care durations have been estimated to save between $55 and $72 million (US) in hospital costs [52]. Ambient AI scribes deployed across extensive clinical encounters have documented savings of approximately 15,700 h in physician documentation time in just one year [53]. In radiology, AI tools have enabled a return on investment approaching 791%, primarily via reductions in radiologist time over a 5 year period [54]. Models optimizing diagnostic test selection via reinforcement learning have demonstrated up to an 85% reduction in testing costs without compromising diagnostic accuracy [55]. The right to health is not blind to distributive justice and goods besides healthcare, and our claims made about AI and the right to health are subject to cost-effectiveness and fairness constraints [56, 57].

Nevertheless, General Comment 14 makes clear that there are certain *minimal core obligations* which States must meet regardless of financial reasonability. These are: 1) the right to freedom from discrimination in healthcare; and, 2) the obligation of States to have a national plan to meet the requirements of the right to health [36]. Thus, States incapable of meeting the requirements of the right to health still have obligations to move toward a future in which they can do so. States ought to take steps to ensure their own future capacity to meet its obligation under the right; present infeasibility is not an excuse [46].

One interesting feature of the human right to health is that the threshold of the minimum core obligation is essentially movable. As new and higher standards of health become feasible and cost-effective, the obligation to meet those standards likewise shifts upward [58]. This is an important point: as a result of scientific progress, moral developments, and increased information, new rights can be articulated to protect against novel threats [14, 59, 60].^5^ That is, there is a moveable threshold of care that is responsive to the infrastructural and technological capabilities that exist at the time. However, how do we know when that threshold has been reached? When did vaccines go from being a recommended course of treatment to something a person has a basic right to access? There are two aspects to this threshold: first, the level of health attainable, and second, obligations in the delivery of healthcare.

The right to health is the right to the *highest attainable standard* of health. This standard is a moving threshold as the human lifespan and healthspan continue to evolve due to technological advances: human life expectancy has almost doubled in the developed world since the nineteenth century [61] and some predictions estimate the maximum human life span will reach 125 by 2070 [62]. In many parts of the world, we have medicine, food security, safe water, reliable accommodation and clothing, and many other goods that form new standards of health. The human right to health thus creates duties on governments to provide sufficient healthcare that can enable each person to reach the modern average human lifespan and healthspan. This standard is not relative: we do not lower our standards of health between developing and developed nations, between metro and rural areas, or between people of different races and ethnicities. We can see this clearly in public health and the right to vaccines: the significant impact of vaccines on human health and their cost-effectiveness at preventing disease renders access to vaccines for common human illnesses a requirement under the human right to health [58], regardless of where one lives. When technology can systemically provide the highest attainable standard of health, particularly in original ways, there is likely to be a claim right to such technology or at least be owed obligations for States to progressively realise such technologies in the future.

Second, there are obligations on states in the *delivery* of healthcare. General Comment 14 sets out four goals that ought to be achieved to fulfil the right to health: measures that secure *available, accessible, acceptable,* and *quality* healthcare (the AAAQ framework). Availability is defined as functioning health-care facilities, goods, and services in sufficient quantity throughout the State according to its developmental level, including the determinants of health, such as safe drinking water, hospitals and personnel. Accessible healthcare requires health facilities, goods, and services to be physically, economically, and informationally accessible without discrimination, with special reference to the most vulnerable and marginalized groups of people. Acceptability refers to the ethical, respectful, and culturally appropriate delivery of health facilities, goods and services. Quality refers to cultural acceptability, and scientifically accurate and medically appropriate facilities, devices, and therapeutics. In recent times, technologies such as the internet have been claimed as necessary under the right to health as it *enables* and *improves* the diagnosis and treatment of medical conditions, enhances the quality, speed, and efficiency of healthcare services, preventative health surveillance, provision of medical literature, and medical education, knowledge, and research [59, 63]. Furthermore, the internet facilitates the availability of healthcare services, such as online mental healthcare, and the accessibility of healthcare to often marginalized and vulnerable groups, such as those living remotely and rurally [63]. When technologies can meet these deliverables under the right to health, particularly when they can meet *multiple* deliverables, such technologies can become essential to provide under the human right to health.

There will be new standards for addressing the right to health if medical AI can significantly increase the number of people who can access healthcare or substantially improve population healthcare outcomes. As we will argue here, this means that States may be required to provide access to medical AI as part of this progressive realization of their duties under the right to health. We do not claim that access to a particular AI tool is the root of the claim to AI (in the same way that the right to internet does not mandate access to a particular search engine such as Google or Bing), but that like the right to internet more broadly, when achievable and justifiable against a State’s existing obligations, that State ought to provide equitable access to efficient or novel AI to help progressively fulfil the requirements of the right to health.

## AI and the right to health

There are three ways AI can address the right to health: (1) improving the quality or quantity of healthcare provided; (2) increasing equitable access; (3) providing healthcare services only achievable by AI. That is, in the same way that many technologies, such as vaccines and medical care during childbirth, have increased the human health span and lifespan and people now have rights to such forms of medical treatment, access to medical AI may similarly be required by the right to health in some circumstances in which it may similarly revolutionize standards of care and improve equitable access to healthcare. Applying the above AAAQ framework, we will argue that when medical AI meets vital needs within public health and in ways incomparable to or more efficiently than other means, then it ought to be permitted, regulated, and progressively introduced through investment and funding. We will acknowledge that such obligations remain context-dependent and subject to considerations of proportionality, feasibility, available resources, and evidentiary support. In this section we will first explain these duties arising from the right to health and explain how medical AI can fulfill such duties significantly *better* than current measures.

### Increased quality of care

Healthcare must not only be available, but it must also meet a certain standard of quality. Indeed, poor quality care may be worse than no care at all as substandard care can lead to misdiagnoses, improper treatment, and other forms of iatrogenic harm. Our claim is not that medical AI ought to be implemented across the board, but only to the degree that its use significantly improves patient healthcare outcomes. Consequently, there is an ethical imperative upon the health system to provide the highest attainable standard of health, one means of which is by using the best available evidence-based practices: such care offers the highest likelihood of achieving optimal patient outcomes. As there is evidence to show that the use of AI tools can provide an increased quality of care, patients can therefore claim that healthcare providers should provide and appropriately use such tools when available, insofar as it fulfills their right to health.^6^ This evidence is abundant in research on the application of AI in healthcare to enable earlier detection of diseases, reduce healthcare costs, and improve patient outcomes. Studies are more commonly showing increasingly successful implementations of AI, including generative AI models such as LLMs and specific biomedical diffusion models (e.g., AlphaFold 3 [64]). AI performs well in areas that require big data 7 due to the capacity of AI to process and analyze massive amounts of data swiftly and effectively, and to uncover patterns that would be extremely difficult to detect manually. It is therefore unsurprising that AI has been shown to excel in diagnostic imaging. Studies have found that AI performs well in radiology [67], cardiology [68], cancer [69], and gastroenterology [70], diagnostics, often matching or even surpassing human diagnostic accuracy [71]. For example, McKinney et al. [72] tested an AI model that outperformed human radiologists in breast cancer screening, with lower rates of false positives (1.2–5.7%, depending on data set) and false negatives (2.7–9.4%). Notably, while human readers had access to patient history and previous mammograms, the AI model was supplied with only the latest mammogram. In addition to normal AI, generative AI has also shown promise. For example, SkinGPT utilizes a fine-tuned version of OpenAI’s MiniGPT-4 model to diagnose skin disease with promising results [73]. While the most salient benefit to the implementation of such AI systems in healthcare would be improved health outcomes for patients, AI systems could also have significant additional benefits with downstream implications for patient care, for example by reducing the workload of human healthcare providers and facilitating medical research. AI systems may also be more cost-effective, for instance, by batching a large number of clinical tasks without a significant reduction in accuracy [74]. In the same way that medical imaging provides better quality and earlier detection of diseases than waiting for symptoms to materialize in more clinically observant ways to the human eye, and is therefore standard in healthcare, so too may medical AI as it continues to evolve and become standardized.

A response might be made here that there are too many instances of medical AI performing only as well as doctors and therefore does not improve current quality of care levels to warrant its requirement under the right to health. In such circumstances, some applications of medical AI may significantly improve the *accessibility* of healthcare by increasing the quantity of healthcare available. For example, medical AI might provide access to more people because of the speed and scale at which AI can deliver services, or increase the capacity of doctors to reach more patients by enabling human personnel to provide healthcare more efficiently [75]. Even if the quality of care is not sufficiently improved, it is generally accepted that adopting tools that do not negatively impact patient care can be justified insofar as such tools are cost-justified [76]. When medical AI significantly improves quality of care or enables standard care to be provided significantly more efficiently and fairly, there is a duty to permit and invest in such AI to partially fulfil the human right to health at a reasonable cost.

### Equitable access to healthcare

The right to health requires healthcare to be *acceptable*: it is a political and international legal commitment to protect the equitable right of each person to achieve a minimally decent life and protect their dignity [42, 77, 78]. General Comment 14 makes explicit reference that the right to health “include the right to a system of health protection which provides equality of opportunity for people to enjoy the highest attainable level of health.” Accessible healthcare is healthcare that is, “within safe physical reach for all sections of the population”. General Comment 14 also interprets a principle of equality and *equity,* that is, healthcare ought:

not disproportionately favour expensive curative health services which are often accessible only to a small, privileged fraction of the population, rather than primary and preventive health care benefiting a far larger part of the population [para 19].

Paul Hunt, the Special Rapporteur of The Right to Health (2002–2008), argues that the right to health “requires an effective, inclusive health system of good quality” [79]. However, many groups of people experience inequality and inequity in access to healthcare, particularly rural and remote residents [80]. This disparity manifests in higher rates of morbidity and mortality, also known as the “rural mortality penalty” [81]. For example, those in rural areas in the United States experience higher rates of chronic disease, particularly in preventable conditions such as obesity, diabetes, cancer, and injury, and are more likely to die prematurely from chronic illness [82]. Rural individuals also engage in risky behaviors at higher rates than urban counterparts, such as substance use and smoking, and lower rates of exercise and nutrient consumption in favour of more calorically dense diets [83]. Those living in rural areas also have higher risks of poor mental health, see for example, the USA [84], across the Asia–Pacific [85, 86], Australia [87], and South America [88]. Many issues contribute to this inequality: financial constraints, scarcity of personnel and services, insufficient or unreasonable transport options, poor internet access, lifestyle-risk factors, health literacy [83], and patients delaying seeking care [89]. The World Health Organization (WHO) attributes health inequality in rural populations to adverse social and environmental determinants, and weaker healthcare systems. In 2016, the WHO estimated that at our current rate, there will be a deficit of 18 million health workers in the attempt to achieve universal health coverage by 2030 [90]. The absence of infrastructure and healthcare personnel in rural regions thus plays a significant role in unequal healthcare outcomes for rural residents. We argue that AI could improve equitable access to healthcare and consider three examples: AI-enhanced remote patient monitoring (RPM), telemedicine, and the use of specialized medical AI in underserved communities.

One example is AI-enhanced remote patient monitoring (RPM). RPM includes the use of wearable sensors, devices, information technology, and telehealth to measure patient vital signs or physiological parameters to assist with clinical decision-making, treatment plans, and general care. With advancements in AI, RPM data can be assessed against medical databases and updated in real-time through machine learning [91]. For example, Iranpak et al. undertook research into a wrist sensor connected to a deep neural network that remotely monitors patient conditions by prioritizing sensitive information in an Internet of Things platform and cloud computing system [92]. The sensor measured heart rate, body temperature, blood pressure, blood sugar, stress, consciousness, pulse, and gait changes.^8^ The remote monitoring system could obtain results with 97.13% accuracy, which was 10.41% better than non-AI methods. Similar successful studies of wearable AI-enhanced sensors, such as smartwatches, or portable devices have been equally successful in detecting cardiovascular diseases [93], cardiac arrhythmia [94], predicting future short-term heart rates and respiratory patterns [95], fall detection [96], monitoring chronic diseases such as diabetes [97], monitoring mental health and predicting suicide [24], prompting timely and accurate dosages for patients personalized from real-time and deep reinforcement learning models based on patient vitals [98], and digitizing personalized adaptive interventions through mobile notifications to the patient to cope with their health problems [99]. AI-enabled telehealth has also been successful in detecting patient vital signs through selecting and analyzing a region of interest on the patient’s face, remotely detecting blood volume changes with a standard smartphone camera, using deep leaning models to recognize patients’ psychological stress levels through video monitoring, and audio monitoring of patients’ coughs to classify the sounds into disease categories [100].

AI-enhanced RPM devices can also provide new forms of care, such as in ‘smart homes’ that feature of several AI technologies to support persons living with dementia. In one case study of such remote monitoring, a client regained urinary continence, increased nightly sleep between 3.5–5.5 h, and reduced “wanderings” at night by 50% [101].^9^ AI-enhanced RPM devices, such as AI-enhanced telehealth, have also been found to reduce travel time, expenses, and the requirement for persons to take forced leave from employment to attend regular check-ups; and can be used to recommend more optimally scheduled visits, as well as increase the number of underserved people who seek and use medical attention [103]. In countries with aging populations, the availability of AI-enhanced RPM can also address an increasing rise in demand on limited resources for quality, efficient, and effective care [91].

AI tools could also provide new and increased *quality* of care for rural patients, potentially in ways that mitigate health inequalities [104]. For example, researchers in Singapore developed an AI model based on convolutional neural networks to diagnose lumbar spinal stenosis (LSS) [21]. LSS is a potentially debilitating condition affecting many adults globally, with a considerable impact on livelihood. Treatment requires diagnosis through a lumbar spine MRI and a tailored treatment plan; however, grading of the MRI can be time-consuming. The AI has been shown to improve scan turnaround time and radiologist productivity. However, where this AI model could revolutionize healthcare would be in the underserved communities, where there are few or no radiologists and spine specialists. Upon early diagnosis by the AI, patients could then be referred to specialists for treatment.

Given the substantial impact AI could have on accessibility in healthcare for historically disadvantaged patients, access to AI could be a requirement to fulfill the right to health in certain contexts. This is because the benefits of medical AI are such that it is already, and will increasingly, redefine what constitutes the “highest attainable standard of mental and physical health”. AI could significantly contribute to the early detection of patient deterioration and early intervention, reduction in hospitalization and rehospitalization, reducing mortality, and provide personalized monitoring of chronic health conditions and mental health resulting in better quality care [91, 100]. AI could transform the way healthcare is provided to and accessed by rural patients [105], and residents have strong reasons to claim access to these transformative technologies. Whilst we acknowledge that there are other ways of meeting the equity requirements of the right to health, such as governments diverting funds from less necessary areas of spending, the unique and complex needs of remote and regional communities are unlikely to *ever* be resourced at levels of means equivalent to those invested in metropolitan areas, due to straightforward economics of scale. Even in countries with public healthcare systems, the difficulties and practical challenges of providing equitable access to healthcare to remote communities may require novel solutions other than building expensive hospitals and personnel, and among these medical AI appears to be especially promising.

### Access to and use of health information

A final example of the relationship between the right to health and medical AI is the impact of medical AI on providing and equalizing accessibility of health information. In the past, healthcare and research were largely dependent on access to physical infrastructures and products such as hospitals, paper records, and medicines. While this is still the case, more recent innovations in advanced medical computing have led to a situation in which a primary driver of healthcare and research is intangible in nature, e.g., data and information [59]. The right to health information is mandated in international human rights law: General Comment 14 states that information accessibility is a determinant of the right to health, namely, “the right to seek, receive and impart information and ideas concerning health issues.” Lemmens and Telfer argue that “access to meaningful information is a critical determinant of the right to the highest attainable standard of health and requires a reliable system of knowledge production” [106]. The right to health information that is *meaningful* thus cannot be isolated from a person’s capacity to *understand* such information. Health literacy and access to reliable health information are thus two closely related determinants of health outcomes, which medical-AI devices could significantly contribute to and improve. We shall discuss each in turn.

Health *literacy* is the capacity of persons to seek, access, and process quality resources, access, and subsequently use health information to improve their health [107] and a critical determinant of health outcomes. Health literacy increases the likelihood of the patient adhering to medical advice, making informed decisions, effectively engaging with health practitioners and in preventative health behaviors, and utilizing healthcare; it also plays a role in reducing their healthcare costs, as well as the likelihood to experience health disparities [108, 109]. Health literacy impacts millions of people; for example, approximately one-third of Americans and 69% of Australians are health illiterate, meaning they lack the ability to access, understand, and use information services to promote and maintain their health [110]. There is substantial reason to believe that AI systems such as specialized LLMs may be used to significantly improve health literacy and thus healthcare accessibility. These models can be used to rewrite and represent information at different levels of literacy and in different styles of delivery [5, 111–113]. AI systems that accommodate patient health literacy can be both actively educational, or substitutive, in that the system undertakes more complex literacy tasks such as translation, simplification, disinformation filtering, and source checking [110, 114, 115]. This allows patients to receive information in their language, or at a literacy level suited to their personal needs. AI can tailor information to the patient’s needs and provide reliable information alongside the reasons *why* such information is reliable, check the patient’s responses for accuracy, and generate multimedia and interactive guides personalized to the patient’s needs. Some research is showing promising results in identifying fake news using hybrid deep learning models with 98.9% accuracy [116]. In an increasingly online and digitized world, even health literate persons should not be expected to hold sole responsibility for wading through the vast amounts of health information and disinformation available online. Medical AI tools that provide the most efficient, accurate, and understandable health information would fulfil the requirement under the right to health to provide people with understandable and reliable medical information without discrimination against health literacy levels.

Medical AI can also provide access to necessary and new health information. Shevchuk et al. argue that in modern society, our health and access to healthcare, “depends on the availability of access to information and digital medical technologies” [59]. They argue that as our knowledge base grows, people have rights to access knowledge that pertains to themselves and their health. Trudo and Telfer argue that “access to reliable information plays a significant role in self-determination and empowerment in healthcare and physical integrity – principles that must surely underlie the notion of the right to health if it is to have any meaningful force and content” [106]. Having access to information about health issues, self-care, and disease prevention increases understanding of personal risk factors, preventative therapies, how to manage their illness, understand their diagnoses, predict their prognosis, cope with disease and understanding when intervention is required, all improves patient health outcomes [107]. Whilst it is difficult to classify all kinds of health information, there are two types of information relevant to the patient that AI models, current and future, may substantially contribute to.

First, AI can generate reliable and accurate *personalized* health information [117]. Various ChatGPT models have been lauded for the significant advantages medical AI can help patients understand medical information, terminology, answer questions, and provide guidance on treatment options [118]. Bentley et al. developed an AI-embedded cyberlearning program that identifies connections between a person’s weight, step count, sleep, location, calendar data, weather, food consumption, mood, and pain, to help person’s identify new and personalized observations that prompt change in health and lifestyle behaviors [119]. Fouad et al. developed an AI-based Internet of Things patient monitoring system that examines patient activities and health status (with 99.86% accuracy) to help the patient choose the appropriate assistance process based on the patient’s future health condition [117]. Varying ChatGPT models have also been used to provide patients with summaries of their clinical interactions, including identification of relevant information, translation of lab results or reports, and to aid patients in managing their health [120]. Finally, AI enables the development of new health interventions that were not previously attainable, such as precision medicine (which involves using genomic analysis and data analytics to create customized strategies for predicting disease progression and treatment responses for individual patients) [23]. Precision medicine therefore concerns, impacts, and facilitates the effectiveness and efficiency of healthcare interventions, by tailoring medical treatment to the individual characteristics of each patient. A diverse array of data is used, ranging from medical health records, genomic, proteomic, and other ‘omics’ or multiomics data, to lifestyle data [121]. As an inherently data-intensive process, precision medicine would be impossible for humans to perform without the support of tools such as machine learning and other forms of AI. AI-embedded devices and programs thus enable people to access new, quality information, and access such information far more conveniently, all of which can help patients and treating teams cost-effectively by early detection and prevention of health problems, and also help patients to better understand their own personal health needs.

Second, AI-embedded programs could also provide *general* medical information for patients such as basic nutrition or exercise advice, preventative health measures, and low-risk treatments for common illnesses. For example, publicly accessible LLMs could be used by people to ask health-based questions. Narula et al. [122] in a study comparing the accuracy of GPT-4 and BERT (Bidirectional Encoder Representations from Transformers) against the accuracy of 4th year medical students, found that whilst LLMs were only 80% accurate in specialized medical queries, LLMs had 88.8% accuracy on *general* wellness, comparable to the students’ accuracy (92.6%). Translational AI models provide means not only for patients to understand their own medical notes, but also for the general public to understand summarized and simplified health information materials such as through freely available LLMs like ChatGPT [110].

These claims nevertheless rest on the capacity of AI to provide medically accurate information [123]. However, we should also be careful as to not expect perfection from medical AI: there are numerous examples of medical technologies that were initially of limited accuracy or reliability, but improved significantly with technological refinement, and iterative development over time to become part of routine healthcare. For instance, earlier prenatal screening methods had much lower sensitivity and higher false-positive rates than current screening methods [124]. Our claims that patients may have legitimate claims to LLMs are thus restricted by conditions of accuracy and quality. We also recognize that generative AI may not compare with specialist medical advice, but it need not replace doctors for it to be efficient, useful, and safe to use in certain circumstances (when it might augment them instead) [75]. The AAAQ framework is pluralistic: healthcare needs to be accessible, and this may be justifiably at the cost of superior accuracy, particularly when much research indicates that medical AI will be as accurate, or is already as accurate, as acceptable physician standards.

The enhanced capability of AI to provide access for people to quality and understandable health-related information is relevant to our understanding of the right to health. The right to health depends in part on the derivative right to access health-based information, not simply freedom to find it if one wishes to: for how can a person look for health information, if they do not know what to look for? The right to health is thus determined both by the patient’s *ability* to access health-related information, but also by whether access to reliable and accurate *information* can be found and discerned. Similar to the internet, as and when AI becomes “an integral part of the information space, as a means of exchanging social information,” people may have legitimate claims to access it [59]. When AI can produce superior, efficient, cost-effective, and quality information in understandable ways to persons of all levels of health literacy, there may be a legitimate claim to it, just as there is in the case of the internet and the information derivable from access to it. Our human rights frameworks thus ought to adapt and accommodate the way new technologies and knowledge modify our understanding of what good quality, efficient, and accessible healthcare means.

## Potential objections

There are several potential objections to our claims. We distinguish here between claims that are unique to the use of AI, and those that apply broadly to the provision of healthcare to satisfy the human right to health. We acknowledge that medical AI can perpetuate issues such as bias, discrimination, and inequitable access, and that these concerns are central when evaluating the impact of AI on health equity.^10^ We will instead deal with specific potential objections to our claim that access to medical AI might be required under the human right to health.

One objection is that AI may undermine, rather than improve, the social determinants of health. Escheverría [20] has considered how AI might undermine the social aspects of the right to health, including by being biased and unexplainable. These are valid concerns. However, they are contingent on assumptions that medical AI will be poorly regulated, and that such intrinsic features are always unjustified. The challenges of medical AI, how to regulate such problems, and what tradeoffs are justifiable, such literature is outside the scope of this paper and has been extensively debated elsewhere [21, 125, 129, 130].^11^ Our case applies only to cases where evidence indicates population health benefits over the status quo (which also suffers from concerns related to accuracy and bias), and in light of technical advances like chain of thought reasoning, tool use (including web search and deep research functionality), the use of fine-tuned and RAG models, as well as ensemble systems, workflows, and multi-agent systems. Current research suggests that imminent gains in accuracy and bias prevention legitimize rather than weaken our claims.

Another objection might be that economic and resource constraints mean AI is not cost-effective to provide. Providing access to AI requires significant investment in technology development, infrastructure and training — resources that not every state would have and it is well recognized that the AI deployment must be located within the circumstances of the application [135, 136]. Consider the previous example of LSS AI. Even if the tool had already been developed by Singapore, if it was to be deployed in a low- or middle-income country (LMIC), the AI will still need to be trained on additional local data for optimized accuracy. However, there may not be sufficient volume of local images to train the LSS AI, and indeed, many LMICs still face hurdles in digitizing health records. In addition, most LMICs lack the resources to develop and implement AI within their healthcare systems. Structural constraints include a lack of funding, lack of expertise, and underdeveloped technological infrastructure [137]. LMICs may lack trained technicians to scan patients with LSS AI and scanning equipment. Furthermore, providing medical AI would demand that resources be allocated to provide access to AI, but why should the state choose to allocate resources to do so when there are other areas in which the resources might be used. We recognize that the right to health accepts structural limitations and that this right is limited by what the State can reasonably provide. However, this does not remove the obligation to provide access to AI to fulfill the right to health in the future or not plan for the progressive realization of medical AI. In the same way that countries must move toward fulfilling the right to health by providing vaccines, surgeries, and equal access to general practitioners, to the extent that AI contributes to these goals, there is a duty to provide medical AI. Even if resources are limited, the provision of AI and other medical tools and therapies is not mutually exclusive: the thoughtful application of AI can be cost-effective and improve health outcomes in settings with resource constraints [50–55, 138]. These duties are nevertheless context-dependent and subject to considerations of proportionality, feasibility, available resources, and evidentiary support.

A third objection might be that the claim ‘medical AI’ is too broad to regulate properly, but it would also be absurd to say that people have rights to specific medical AI models. In the same way that people do not have a right to specific brands of treatments, e.g., Panadol as a brand of paracetamol, people do not have a right to ChatGPT-4, or some other forms of AI-embedded healthcare. However, the right to AI is not to be used to demand rights to specific brands of drugs, but to demand access to a class of treatments, such as antibiotics or over-the-counter pain relief. In the same way we do not require people to have access to particular brands of vaccines but nevertheless have rights to standardized and effective vaccines, we believe that people have access to AI-embedded devices and platforms to achieve the goals *only* AI can achieve (such as personalized medicine), or the goals that AI can achieve in the most efficient, accurate, and cost-effective means. That is, insofar as medical AI uniquely meets the requirements of the ‘highest standard of health’ under the UDHR Art. 25 by significantly improving the acceptability, accessibility, or quality of healthcare, there is a claim right to medical AI, or that governments ought to progressively realise a future in which such access can be guaranteed as of right. There is flexibility in our argument because it is not a right to a *specific* AI tool. We argue that fulfilling the right to health is partially dependent on access to medical AI is a claim embedded within the broader right to health, so if access to AI can help achieve the provision of quality healthcare, then access to AI ought to be provided. For example, if there is a lack of radiologists and spine specialists in rural communities, and the AI to diagnose LSS (and associated requirements such as equipment and training for technicians) is available at a reasonable cost, then that tool ought to be provided.

Another objection is that the implementation of medical AI in the way recommended in this paper may be impractical or imprudent. More specifically, some scholars have raised concerns that telehealth can widen health disparities between urban and rural patients due to poor internet or technology access [139]. Other concerns arise in relation to the storage and protection of patient data in the absence of end-to-end encrypted communication devices [140]. These are legitimate worries; indeed, inequalities within and between States means that there are differential obligations under the right to health and many countries can only be expected to progressively realise such rights. Furthermore, the right to health is not blind to other rights, such as the right to privacy, nor is it blind to the social and unexpected outcomes that might arise from the use of medical AI in practice. As with the implementation of any positive right, investing and providing access to medical AI to partially fulfil the right to health will require active regulation and monitoring of unintended and harmful consequences, such as overuse or misuse and privacy breaches. However, that the implementation of medical AI in healthcare will at times be practically difficult and require careful balancing of values and competing rights is not an impediment to the claim that technologies that significantly improve efficiency in, and access to, healthcare are essential in partially fulfilling the human right to health. Indeed, this is a concern that arises in the implementation and politics of all human human rights.

A final objection is that the use of medical AI, particularly for efficiency reasons, may undermine the quality of care received by patients. For example, some authors have raised concerns with telehealth, given its overuse or misuse can increase costs to providers although scholars have recognized this is partly a regulatory matter and not inherent to the nature of telemedicine [141]. Other worries arise on the basis that implementing AI into healthcare will increase burdens on healthcare professionals who must spend significant amounts of time learning and maintaining such systems [142]. This leads to another worry that the improved efficiency as promised by medical AI may only be achieved if clinicians spend less time with patients, resulting in a higher number of patients seen during a certain time period but potentially with a lower level of care [142].^12^ Kempt, Freyer, and Nagel have raised concerns about how adopting medical AI as a means to provide access to healthcare lead to different standards of care between communities and even within hospitals [143]. Daus has argued that using medical AI to provide healthcare access to remote communities might simultaneously reduce patient self-respect through mis-recognition, particularly through racial bias [144]. Furthermore, some governments may lack the capacity to collect sufficiently robust local datasets or to train the AI tools, potentially exacerbating existing inequities in access and performance. The massive amounts of data required to develop beneficial and accurate AI at the core of these objections may open patients up to privacy and confidentiality issues, particularly when such data is controlled by tech companies or governments.

These are legitimate worries. We acknowledge that the duty to permit and invest in novel AI only arises insofar as these technologies actually *improve,* rather than worsen, efficiency of providing overall healthcare, access to healthcare, or healthcare outcomes. Nevertheless, these are structural and regulatory challenges regarding the practical implementation of medical AI in healthcare. Whether healthcare practitioners receive support is a structural issue, such as through paid introductory education and training, or the provision of AI technicians equivalent to existing laboratory technicians. Similarly, governments can actively ensure clinical or regulatory validation of individual AI tools, in equivalent ways to the validation of AstraZeneca or Pfizer are examples of safe and effective vaccines, through regulatory and legal decisions to oversee these tools. Whether and how patient data is protected and used is also a regulatory choice, rather than a presumptive default. Indeed, in order for medical AI to justifiably be used to partially fulfil the right to health, governments must actively regulate and install infrastructure to protect other rights, such as the right to privacy and confidentiality.

Furthermore, to the degree that the costs of healthcare services must be justified against the costs, some reductions in standards of care may be justified in some limited circumstances. For example, funding an expensive but accurate treatment of a rare condition may be justifiably sacrificed in favour of a moderately costly treatment of a rare condition to enable some funding to be simultaneously invested in a condition suffered by many people. Some compromises to quality of care may be made in the introduction of medical AI insofar as we do not have other practical, cost-effective, or equally beneficial options in achieving equity in healthcare access [133]. It is reasonable that *slight* reductions in quality of care can be legitimately justified in some circumstances, particularly when significantly more people gain access to sufficiently beneficial care to which they wouldn’t otherwise have access [76]. However, we endorse the view that determining when a tradeoff between rights, or between values such as beneficial outcomes and the need for human interaction, ought to be determined with effective, culturally appropriate, and genuine democratic deliberation [144, 145].

## Balancing and proportionality in the medical AI context

Throughout our analysis, we have acknowledged various legitimate concerns about requiring medical AI under the right to health: that such technologies may be prohibitively expensive, accessible only to privileged populations, environmentally costly, dependent on extensive digital infrastructure, or in tension with other fundamental rights such as privacy. A particularly salient objection is that States should prioritize establishing basic healthcare infrastructure and expanding traditional healthcare access rather than investing in medical AI, especially when many populations still lack access to clean water, basic medicines, and primary care services. While we acknowledge the critical importance of these foundational elements of the right to health, we respectfully submit that this framing presents a false dichotomy. The obligation to progressively realize the right to health does not require States to choose between traditional healthcare infrastructure and medical AI; rather, it requires them to pursue all cost-effective means of advancing the highest attainable standard of health. Indeed, as our analysis of efficient AI demonstrates, medical AI may itself be a mechanism through which States can more effectively provide traditional healthcare services. For instance, by enabling remote diagnostics in underserved areas where building physical infrastructure remains prohibitively expensive or logistically challenging.

The right to health, as articulated in Article 12 of the ICESCR and elaborated in General Comment 14, does not exist in a vacuum but functions within a sophisticated system of balancing and proportionality. Indeed, the obligation to provide the "highest attainable standard of physical and mental health" is inherently subject to both the principle of progressive realization and the legitimate limitations outlined in Article 4 of the ICESCR, which permits restrictions "determined by law only in so far as this may be compatible with the nature of these rights and solely for the purpose of promoting the general welfare in a democratic society." Importantly, our claim that medical AI may be required under the right to health is conditional, not absolute. We are not suggesting an absolute or immediate obligation that overrides all other considerations. Nor are we suggesting that AI should divert resources from basic healthcare needs. Rather, states must engage in a genuine proportionality analysis that weighs both benefits and risks, considering medical AI alongside other healthcare investments.

This proportionality requirement stands in marked contrast to current regulatory approaches, particularly the EU AI Act, which adopts a predominantly monistic, risk-based approach [146]: unacceptable risks, high risks, and low or few risks (which must comply with few or no requirements) [147]. A key factor of the EU AI Act, is that deployers of high-risk AI systems must assess the extent to which an AI device impacts fundamental rights [147]. This approach has been subject to criticism, particularly insofar as it systematically overlooks countervailing benefits. Ebers [148] argues that the EU AI Act lacks a ‘risk–benefit’ analysis: the Act only balances the potential to cause harm with the severity of the likely harm and thus fails to balance the risks and harm against potential benefits of technology within the regulatory framework. Such an approach risks creating what might be termed a regulatory asymmetry: while potential harms from bias, privacy breaches, or misuse are extensively catalogued and regulated, the potential for medical AI to dramatically improve healthcare access for underserved populations, enhance diagnostic accuracy, or enable entirely novel therapeutic interventions receives insufficient – indeed, no – weight. For example, Ebers argues that the risk-based approach fails to strike an appropriate balance in healthcare, in which it is unclear whether medical AI devices ought to have a minimum level of transparency before being released on the market. The trade-off between transparency and accuracy is missed when the benefits of accurate AI cannot be considered in a test of proportionality. Whilst some scholarship has analysed the ways in which the EU AI Act undermine rights in relation to patient care, such as the right to an explanation, the right to withdraw from, or context, AI decision-making, and the right to a second opinion [1], the failure to balance the benefits of AI in a regulatory approach allows for the exclusion of the ways in which medical AI could support existing fundamental rights, such as the right to health.

This approach is fundamentally inconsistent with the balancing methodology required under international human rights law. As General Comment 14 makes clear, the right to health requires states to ensure that healthcare is not only safe but also available, accessible, acceptable, and of good quality. If medical AI can significantly advance these dimensions — for instance, by making specialist diagnostics accessible to rural populations through AI-enhanced telehealth, or by enabling personalized medicine approaches previously impossible — then these benefits must be weighed against legitimate concerns about cost, privacy, and equitable distribution. The progressive realization principle does not excuse states from this balancing exercise; rather, it requires them to move "as expeditiously and effectively as possible" toward full realization while taking into account their available resources and competing obligations. Our argument is a conditional one: when medical AI demonstrably advances the highest attainable standard of health in cost-effective and feasible ways, states cannot categorically exclude it based solely on risk considerations alone. Human rights law requires that such exclusions be preceded by genuine proportionality analysis.

A further dimension of this proportionality analysis that warrants explicit attention is the obligation of justice. In balancing the benefits of AI with the harms it may pose, we must consider both whom is benefited and who is harmed [149]. Principles of egalitarian justice, such as John Rawls’ [150], would raise concerns if the harms of medical AI actively worsen outcomes for those already subject to structural disadvantage. For instance, a system that improves diagnostic accuracy for patients of European ancestry while performing worse for patients of African ancestry, or that streamlines consultations for articulate, dialect-free speakers while disadvantaging those with accents or speech difficulties, or that enhances access for smartphone-literate patients while creating new barriers for the digitally excluded, cannot straightforwardly be said to advance the right to health.^13^ Under international human rights law, the right to health is explicitly non-discriminatory, and General Comment 14 identifies vulnerability and marginalisation as factors demanding heightened rather than diminished attention. We therefore submit that the proportionality analysis required of states must incorporate some consideration of equity: it is not sufficient that aggregate outcomes improve if those left worse off are drawn disproportionately from groups already bearing the heaviest burden of structural injustice. However, this is a principle which applies to all health care, not only AI. Whether such a trade-off is ultimately acceptable will be context sensitive. For instance, whether a non-AI alternative remains available, the magnitude of harm to the disadvantaged group, the degree to which existing care is already inequitably distributed, and whether the disadvantaged group will actually be made worse off, or simply not equally as better off as already advantaged groups. We do not seek to balance these claims for all contexts, indeed we cannot, but we do believe that the moral weight of the magnitude of benefits of medical AI in particularly fulfilling the human right to health renders its use morally salient and ought not to be forgone simply due to the existence of any inequality.

## Implications of AI as required by the right to health

Our overarching claim has been that insofar as medical AI supersedes existing means to effectively, equitable, and originally improve healthcare access and outcomes, access AI-embedded devices or platforms is progressively required under the right to health. We have distinguished between efficient AI, namely medical AI that makes existing healthcare services significantly more efficient thus increasing the cost-effectiveness of healthcare services, and novel AI, namely medical AI that provides novel and original solutions to existing healthcare problems. To the extent that novel or efficient AI significantly improves healthcare outcomes, or improves access to healthcare, there will be a corresponding duty to progressively realize its research and implementation in healthcare. Importantly, this obligation is a conditional one: dependent context specific proportionality analysis. This claim has implications for States and their obligations towards people within their jurisdiction. We see two main obligations on governments to ensure they are not in violation, now or in the future by failing to progressively achieve these obligations, of the claim to AI through the right to health [59].

First, there is an obligation on governments and regulatory bodies to regulate the implementation of AI to not unnecessarily restricting access through regulation, access to medical AI ought to not be unnecessarily restricted by excessive regulation and prohibition. Furthermore, States ought to regulate in ways that both encourages the innovation and use of AI in healthcare systems but also protects patients from additional harms that AI creates. This is a fine balance: the right to health and the claim to AI may depend on accepting new risks in the breach of other rights, such as the right to privacy and confidentiality. Nevertheless, there are many resources and research presenting frameworks as to the best means to regulate without stifling the use of AI in healthcare [151].

Second, there is an obligation on States to spend resources in making AI-embedded healthcare available when affordable and reasonable for the State to do so. This requires investing in research and organizations that provide and improve AI-embedded healthcare programs and devices. General Comment 14 requires States, “[d]epending on the availability of resources… [to] facilitate access to essential health facilities, goods and services in other countries, wherever possible, and provide the necessary aid when required.” In the same way that States ought to make drugs, therapies, hospitals, software systems, doctors, and essential aspects of healthcare affordable, if not fully subsidized, States have an active duty to make AI-embedded devices and programs similarly affordable or subsidized. This also requires States to provide AI-embedded platforms and databases that provide reliable and accurate health information. General Comment 14 paragraph [16] prescribes that States *must* provide access to education about prevention, treatment and control of diseases and that this requires States to make available relevant technologies to satisfy the right to health. We nevertheless acknowledge that providing equitable access to AI-enabled healthcare faces significant structural barriers, especially in lower-resourced settings. These include limitations in funding, technological infrastructure, and digital health capacity [137]. For example, many health systems still struggle to digitize health records, a foundational prerequisite for AI implementation [138]. The effective deployment of AI in healthcare often presupposes access to digital infrastructure such as the availability of devices, reliable internet connectivity, and stable power supply. This concern is reflective of broader structural difficulties in balancing values within the human right to health, as well as against other human rights, such as to clean environments. For example, it would be difficult to justify the use of medical AI that can slightly improve the outcomes of people in industrialized states, but does so at the cost of freshwater availability in areas with more vulnerable populations. For this reason, we understand the right to AI in health as one that must be realized progressively, sensitively to context, and in conjunction with competing rights. This framing recognizes that while AI may offer novel and effective ways of fulfilling the right to health, its implementation must not undermine other fundamental rights or exacerbate existing global health and environmental inequities.^14^

Furthermore, in the instance that a State *cannot* invest or produce such resources itself, General Comment 14 in its paragraph 42 states that “States parties should refrain at all times from imposing embargoes or similar measures restricting the supply of another State with adequate medicines and medical equipment.” As Muyskens has argued, in instances where States are not themselves capable of fulfilling the right to health (for example, because they are unable to provide or appropriately make available medical AI), there is an obligation on the part of these States to seek—and on other States to provide—international assistance towards the fulfilment of this right [30]. In the context of AI-embedded platforms, therefore, this may only require other states to make AI-information platforms globally accessible and prevent the prohibition of access by international citizens on medical AI platforms rather than physically intervene to provide such AI programs and infrastructure [46].

## Conclusion

We have argued that the if the promise of advanced and superior quality and efficacy of medical AI can fulfil the right to health in comparison to current medical measures, there will be a conditional requirement on states to provide access to those medical AI systems, or at least make plans to provide access to such medical AI in the future. Since medical AI can fulfil some of the requirements of the right to health, medical AI ought to be regulated in such a way that does not stifle innovation or unnecessarily restrict its widespread use, yet also ensures safety and accountability. These requirements are subject to evidence of AI quality or contributions to efficiency, resource availability, and context-specific ethical considerations.

## Data Availability

No datasets were generated or analysed during the current study.

## Footnotes

1 Additionally, the invention of new technologies can also raise questions about the creation of new rights. For example, Kim and Routledge argue that the use of AI creates a new right to an explanation [16]; and others have argued that the human right to enjoy the benefits of the progress of science and its applications (the ‘right to science’, see [17]) may, when applied to AI, justify a right to universal basic income [18]. Whether there is a separate right to AI, alongside existing traditional human rights that address fundamental needs (e.g. food, shelter, education, health) and freedoms (e.g. speech, privacy), is a distinct and separate issue, and considerably broader than the context of health alone. Given there is an existing right to health, we wish to examine how current and future medical AI technologies impact, and are impacted by, this existing right. We are not arguing that there is a separate right to medical AI. Rather, we are making the narrower claim that because medical AI can fulfill the right to health better than current measures in certain contexts, access to AI may be required to fulfill the right to health.

2 It is outside the scope of this paper to argue how the right to health ought to be balanced against other existing, and often interpreted as more pressing, rights. Indeed, this paper should not be seen as a call to divert funding from these rights, but as a way to prioritise within healthcare systems. For example, Larry Temkin [ 25] has argued that insofar as addressing the social determinants of health, such as education, income, and environment, can more cost-effectively prevent disease than investing equivalent funding in reactive healthcare treatments, there may be obligations to divert funds to already healthy people despite the urgency of existing patients.

3 See, for example, prudential reasons [26], agency and autonomy [27], basic needs [28], capabilities and positive freedom [29, 30], dignity [31]; and fairness, status equality and equal respect [32].

4 There is still opposition to normalizing and implementing the right to health in domestic law, and, separately, in philosophical literature. This is because the right to health is commonly seen as a positive right, that is, protecting the right to health requires States to *spend* resources on healthcare, personnel, infrastructure, research, and subsidies. However, some scholars reject the legitimacy of positive rights in favor of negative rights on the grounds that positive claim rights are not fulfillable and therefore no duty is owed [40]. They argue that the only rights that States are required to protect are negative rights, namely, political and civil rights as States only need to pass law prohibiting such actions, given such actions can be taken with minimal resources and protect individual liberty. Yet for those who accept the legitimacy of State spending on socioeconomic and cultural rights, the right to health fits squarely within the jurisdiction of States. Many scholars now accept that such a dichotomy between positive and negative rights is either unclear, or irrelevant [34, 40, 41]. For example, Faden and Beauchamp argue that “it is possible that positive obligations to provide benefits may flow from negative rights’ [42]. Similarly, Evans argues that the liberal claim that negative rights “are free of cost overlook the necessity for state intervention in the name of protecting human rights” [40]. What matters, therefore, is the *scope* of the right.

5 While there is no definitive list of new rights, there are proposed themes. See for example, Baroni [60] and Risse [14]. Risse argues we have negative rights against interference from digital technologies such as breaches into our privacy, confidentiality, and misuse of our data, but also positive rights to digital phenomena like information derived from the mass accumulation of data.

6 While we acknowledge that international law does not presently recognize any general duty to provide novel technologies, the principle of progressive realization and the obligation to ensure adequate infrastructure suggest a trajectory in which states should incorporate proven technologies, including AI, as part of fulfilling the right to health.

7 Big data refers to vast and complex datasets that are challenging to process, store, or analyze using traditional data management tools [65]. It is often characterized by ‘3 Vs’: volume, variety, and velocity, though other ‘Vs’ such as variability, veracity, value and visualization have also been highlighted [66].

8 Although the paper reports measuring consciousness, some of the authors have concerns that consciousness can be measured remotely at all and follow research that suggests consciousness can only be measured in first person.

9 Although the authors [101] use the term “wanderings”, we wish to acknowledge that this term has implicit normative judgment, which is often used to stigmatize persons living with dementia. However, we wish to also recognize the risks persons living with dementia face due to a condition-associated increase in prevalence of activities undertaken in dangerous or unsafe ways. See Chelberg for a detailed analysis [102].

10 A worry might arise here that the use of medical AI might *exacerbate*, not merely perpetuate, the harms arising from the use of biased medical data. This concern is extensively documented and examined in the literature [7, 125–128]. It is important to clarify, however, that our argument is specifically normative, asserting that medical AI, if it demonstrably provides significant healthcare benefits and supports equitable resource distribution, may become required under the human right to health. AI outweighs these benefits, and how best to regulate or mitigate such harms, involves detailed contextual analysis and practical regulatory considerations that are beyond the scope of this paper.

11 See London [131], Prince and Lim [129], Babic and Cohen [132], and Cohen [76] for debates about the ethics of black-box AI in perpetrating harms in patient care, and Prince and Savulescu [133], Vandersluis and Savulescu [134], Acquino et al. [125], and Muyskens et al. [21] for discussions on the justifiability of using biased black-box AI in healthcare.

12 We would like to thank one of the reviewers for raising our attention to this paper and concern.

13 We are grateful to our anonymous reviewer for raising these points and examples.

14 The difficulties and challenges in balancing the human right to health and other rights, such as the right to privacy and right to a clean environment, or right to water, have been extensively documented. See for example, within the context of Covid-19 and the environmental harms of health infrastructure waste [152], and the difficulties of balancing the right to vaccinations against the right to autonomy [153]. It is outside the scope of this paper to balance the right to health with other rights, or the various means of fulfilling the right to health. We only argue that a new option on the table of fulfilling the human right to health arises with the invention of significantly more effective or novel medical AI.
